# Habits, Traditions, and Beliefs Associated With the Use of Complementary and Alternative Medicine Among Diabetic Patients in Al-Qassim Region, Saudi Arabia

**DOI:** 10.7759/cureus.33157

**Published:** 2022-12-31

**Authors:** Ebtehal S Almogbel, Fai M AlHotan, Yazeed A AlMohaimeed, Majd I Aldhuwayhi, Saud W AlQahtani, Sultanah M Alghofaili, Barah F Bedaiwi, Aswar H AlHajjaj

**Affiliations:** 1 Department of Family and Community Medicine, College of Medicine, Qassim University, Buraydah, SAU; 2 College of Medicine, Qassim University, Buraydah, SAU

**Keywords:** type 2 diabetes mellitus (t2dm), type 1 diabetes mellitus (t1dm), dm-related characteristics, saudi arabia, beliefs, traditions, habits, alternative medicine, complementary medicine

## Abstract

Background

Since diabetes mellitus (DM) affects every aspect of a person’s being, more and more people are using complementary and alternative therapies such as ingesting ginger and cinnamon in addition to conventional medical care and lifestyle changes to manage their condition and enhance their well-being. Although this population uses complementary and alternative medicine (CAM) at a high rate, it is unclear what causes this use.

Objective

We aim to know the habits, traditions, and beliefs associated with the use of complementary and alternative medicine among type 1 diabetes mellitus (T1DM) and type 2 diabetes mellitus (T2DM) patients in the Al-Qassim region of Saudi Arabia.

Methods

This is an observational cross-sectional study conducted among diabetes patients in Al-Qassim, Saudi Arabia, in 2022. Participants were selected via a non-probability sampling technique. Patients were interviewed in the diabetic clinics using validated questionnaires. Data were analyzed using the Statistical Package for the Social Sciences (SPSS) software (IBM SPSS Statistics, Armonk, NY, USA).

Results

A total of 444 validated responses were received in this study. The average age was 50 ± 16.9 years, and females represented the highest proportion (58.6%). Moreover, we found that most of the participants had type 2 diabetes (79.1%) and 93 (20.9%) had type 1 diabetes. Hypertension was the most reported chronic disease. Our results revealed that the prevalence of CAM usage was 29.1%. Regarding the sources of information on herbal medicines, we found that more than half of the respondents (57.4%) obtained information from friends, relatives, and neighbors. Ginger, vitamins and minerals, and cinnamon were the most frequently used herbals among our participants. Our results found that 38% of CAM users used herbal products on a regular basis. As regards the frequency of using herbal products, 29.5% of the respondents used herbal medicine weekly and 21.7% used it daily. In addition, we found that gender, marital status, and monthly income were significantly associated with the use of CAM (P value = 0.008, 0.011, and 0.011, respectively). The significantly higher CAM use was associated with females, married participants, and participants with a monthly income of 10,000-15,000 Saudi riyal (SAR).

Conclusion

According to our research, CAM use among diabetes patients in the Al-Qassim region was found to be relatively common. The prevalence of type 2 diabetes mellitus was higher (79.1%) in comparison to type 1 diabetes mellitus (20.9%). Also, the most commonly used herb was ginger (47.66%), followed by vitamins and minerals (44.53%), and cinnamon (42.19%). Patients with diabetes need to be informed of the significance of telling their doctors about their use of CAM.

## Introduction

Herbs have been used as a treatment for various medical problems since ancient times, and some people still prefer to try and use them for treating a variety of health issues. However, while herbs can help improve health and overall well-being, they have some drawbacks; for example, some Chinese herbs can cause serious side effects such as acute kidney injury, chronic kidney disease (CKD), nephrolithiasis, and rhabdomyolysis [1].

Moreover, herbs can interact with medications, such as prickly pear cactus with glipizide, resulting in hypoglycemia, and *Allium sativum* with chlorpropamide [2,3]. Furthermore, because herbs are not prescribed by physicians or professionals, it is impossible to know the exact dose to be taken. Therefore, patients must consult their treating physicians before beginning to use it.

Diabetes mellitus (DM) is a serious and widespread chronic disease affecting people of all ages, genders, and socioeconomic backgrounds in Saudi Arabia, with 2,156,294 patients out of a population of more than 30 million. Diabetes affects 80,739 people in Al-Qassim specifically. Type II diabetes accounts for the majority of diseased people (90%) [4]. Patients with type 2 diabetes are treated with oral hypoglycemic medications such as metformin, which reduce insulin resistance by decreasing hepatic gluconeogenesis and increasing insulin sensitivity. When it comes to the management of DM, there are several approaches. Some of them recommend taking certain herbs, such as ginger and cinnamon, instead of or in addition to prescribed medications.

Our aim in this study is to evaluate the prevalence of those practices in Al-Qassim, Saudi Arabia, and to understand diabetic patients’ awareness regarding their illness, medication, and their herbal use. Additionally, we will investigate more the type of herb they mostly used to treat DM or decrease blood sugar levels.

Diabetes mellitus affects people of all socioeconomic levels worldwide, including Saudi Arabia. Saudi Arabia ranks second in the Middle East and seventh globally in terms of diabetes prevalence [5]. This is considered to be a burden on own personal health, national health, and economic level. As a result, Saudi Arabia is attempting to aid diabetic patients by, first, providing free treatment; second, making home visits to patients who live a long distance from health services; and, third, offering discounts on home diabetic screening devices and supplies.

This chronic disease affects nearly all the body’s functions, resulting in long-term complications such as retinopathy, nephropathy, neuropathy, and diabetic cardiomyopathy. Its short-term life-threatening complications include diabetic ketoacidosis, hyperosmolar hyperglycemic state, and hypoglycemia. It also has a considerable effect on the patient’s life quality and capacities.

Diabetes mellitus in general has many types, depending on the pathogenesis and the time of incidence, starting from the impaired secretion of insulin (type I) to impaired utilization of the produced insulin (type II), and the management approach differs in each patient according to the type, dosage, number of medication according to the type of DM, patient response, and caloric intake, along with other factors to be considered.

The main interest of this study, considering the high prevalence of type 2 diabetes globally and in Saudi Arabia specifically [5], resulted in a variety of treatment approaches for this chronic disease, for instance, complementary medicine, where people ingest certain herbs to lower blood glucose levels in adjacent to their prescribed medications, or alternative medicine, in which patients fully depend on a different approach of treatment other the standard medical treatment [6].

Based on published literature, there is a considerable percentage of diabetic patients who aim to reduce their blood glucose levels with various herbs, and we hypothesize that there is a high prevalence of herbal use in the Al-Qassim region.

An observational prospective cross-sectional study was done in 2019 and conducted in a tertiary care hospital in Pakistan to identify the use of complementary and alternative medicine (CAM) products among diabetic patients. The results showed that 151 (57.8%) patients used complementary and alternative medicine. The most commonly used CAM techniques were herbs (n = 121, 80.1%), special diets (n = 98, 64.9%), and cupping (n = 68, 45%). Female gender, older age, unemployment, longer duration of diabetes, diabetes-related comorbidities, and poor glycemic control were all linked to CAM use [7].

In addition, a descriptive comparative study of DM patients in western Algeria that was conducted in 2021 showed that the use of herbs among diabetic patients was reduced compared to previous studies. Patients taking oral hypoglycemic agents are at twice the risk of combining them with herbs. The most common herbal seeds used by the patients were *Trigonella foenum-graecum* seeds and *Olea europaea* leaves. Many clinical studies have shown that antidiabetic plants have a synergistic effect with oral antidiabetic drugs and may increase hypoglycemia in these patients [8].

Another cross-sectional study was conducted among diabetic patients in Ethiopia in 2018 with 387 respondents, of whom 62% were herbal users. The most common herbal medicines were garlic (41.7%), Giesilla (39.6%), Tinjute (27.2%), and Kosso (26.9%). The majority of herbal medicine users (87.1%) did not tell their doctors about their use. Their sources of information on herbal use are family and friends (51.9%) and DM patients who also use herbs (28.9%) [9].

Moreover, a mixed-methods study using descriptive statistics that included a survey administered by the investigator as well as focus group discussion sessions was done in 2018 to identify the prevalence, beliefs, and behavior linked to the usage of alternative medicines for hypertension and type 2 diabetes mellitus (T2DM) in Jamaica. Of the respondents, 87%-90% were on prescription medication, 69% said that they took their medication exactly as recommended, and 70% said that it helped them regulate their symptoms. Furthermore, 98% said that they used alternative treatments, mostly herbal medications, and 73%-80% said that herbs have helped them regulate their symptoms. Herbal medicines, according to one-third of the respondents, are the most effective form of therapy and should always be used rather than prescription drugs. According to 85% of the respondents, prescription and herbal medicines should not be utilized at the same time. The majority (76%-90%) did not tell their doctors about their herbal treatments [10].

A cross-sectional study was done in February 2022 to determine the prevalence and types of herbal medicine in diabetic clinics in Kuwait. A total of 350 patients completed the questionnaire. Of the participants, 30.6% use herbal supplements, with women being more inclined to do so than men. The most commonly used plant among diabetics was black cumin (habba soda), also known as *Nigella sativa*. According to the study, 70% of diabetics who use herbs have poor blood glucose control (HbA1c of less than 7%). Patients who received herbal treatment experienced higher diabetes complications than those who received standard care. Of the herbal medicine users, 95.3% did not inform their doctors that they were taking the medication [11].

Another study was also conducted in the United Arab Emirates (UAE) in 2020 that included 244 patients with type 2 diabetes (response rate: 80%). Of the respondents, 39.3% have been CAM users since diagnosis. After data validation, logistic regression results showed that CAM use is strongly associated with age (56.9 ± 13.3), gender (female: 66.7%, male: 33.3%), education (primary education: 40.6%, secondary education: 50%, higher education: 9.4%), employment (employed: 43.8%), and health insurance (with health insurance: 81.3%). The most common types of CAM among participants were herbs and folk foods, followed by natural remedies, mental health supplements, vitamins, and minerals. Most CAM users were recommended to use it by their family and friends (42.7% and 25%, respectively) or social media (17.7%). Only 13.5% used CAM as recommended by their doctor, and only 25% of CAM users talk to their doctor about using CAM [12].

A descriptive review was done in 2017 to identify the prevalence and the most used therapies of traditional and complementary medicine (T&CM) among diabetics in Saudi Arabia. The result showed that T&CM was used by 32.18% of diabetics in Saudi Arabia. Herbal medication was shown to be the most commonly used T&CM therapy among diabetics. Fenugreek, black seeds, neem, myrrh, helteet, harmel, and aloes were the most commonly used herbs [13].

Moreover, a descriptive cross-sectional study was conducted among diabetes patients attending primary health clinics in the National Guards and Military Hospitals in Taif, Saudi Arabia, between February and June 2019. The result showed that the prevalence of complementary and alternative medicine use was 33.7%, 87.3% did not consult a doctor before using CAM, 43.2% had more than one source of information, and 62% used more than one complementary and alternative medicine approach. Around 49.2% said that it was very useful, and 72.9% said that it had no negative side effects. Furthermore, 47.5% of the diabetic patients would recommend complementary and alternative medicine to other diabetic patients. Patients reported using bitter apple (100%), cinnamon (66.1%), ginger (55.1%), fenugreek (35.6%), and garlic (21.2%) as their only complementary medicine. Family history, female gender, diabetes complications, and having diabetes for a long period of time were all associated with increased use of complementary and alternative medicine (CAM) [14].

Between January and March 2019, a cross-sectional survey was done in several hospitals and medical centers in Makkah, Saudi Arabia. A total of 289 type 2 diabetes patients and 105 physicians were interviewed. Of the participants, 68% used herbal treatments regularly, especially cinnamon, ginger, and fenugreek. Family and friends, as well as social media, were the primary sources of information on herbal use among the patients. The majority (71.4%) did not consult or inform their doctors about their decision to self-medicate with herbs. Patients were unconcerned about the efficacy and safety of herbal medication in the treatment of diabetes. Almost half of the participants believe that herbal therapy is both effective (54%) and safe (46%) for treating diabetes symptoms. Two-thirds (66%) of doctors ask patients if they use herbs to treat their conditions regularly. Of the doctors, 25% were enthusiastic about herbal medicine in the treatment of diabetes, while others were concerned about the increase in herb use and wanted to see further attention paid to safety issues [15].

## Materials and methods

Study design, area, population, and sampling

This is a face-to-face interview-based observational cross-sectional study conducted in Al-Qassim, Saudi Arabia, that included 444 diabetic patients from August 1 to October 31, 2022, to know the habits, traditions, and beliefs associated with the use of complementary and alternative medicine. In this survey, patients were interviewed in diabetic clinics and primary care centers in Al-Qassim using validated questionnaires. Those who came for regular follow-ups to check blood sugar levels and refill medications during the data collection period were considered the study population. Those with T1DM and T2DM were included in this study. Those without DM are excluded, as well as those with T1DM and T2DM but live outside Al-Qassim.

We used the Epi Info software for the calculation of the required sample size based on the following assumptions: total population size of diabetic patients in Al-Qassim = 80,739 [4], expected frequency = 50%, acceptable margin of error = 5%, confidence limits = 95%, and design effect = (1). The calculated sample size was 383.

Methods for data collection

The predesigned structured questionnaire used in this study was made out of two validated questionnaires of similar articles [12,14]. The questionnaire was originally written in English and was then translated into Arabic (since all of the patients spoke Arabic). A professional translator back-translated the translated Arabic version to ensure the parallel form reliability of the questionnaire, to ensure that all questions were properly translated, and to check the translation quality. The survey instrument was divided into six parts: sociodemographic characteristics, diabetes-related characteristics, prevalence of herbal usage among diabetic patients, herbal-related characteristics among herbal nonusers, information about herbal medicine, and herbal-related characteristics among herbal users.

The first part of the questionnaire collected sociodemographic data that included age, gender, nationality, marital status, monthly income, educational level, and occupation. The second part of the questionnaire included questions related to DM characteristics, such as if they have any other chronic diseases, age of diagnosis of diabetes type 2, duration of diabetes mellitus, family history of diabetes mellitus, presence of diabetic complications and cerebrovascular diseases, and type of medication they are taking. The third part of the questionnaire focused on CAM use. In the fourth part of the questionnaire, we asked patients if they believed in CAM, where they obtained their information, and why they used herbal medicine. The fifth part of the questionnaire asked about the name of the herbal medicine, if they take it regularly, the period of time over which they use it, the reasons for using CAM products, how they feel after taking the products, if there are any adverse effects associated with them, if they tell their doctor about their use of CAM products to control diabetes, would they recommend the treatment to other diabetics, and if they would consider using CAM in the future.

Statistical analysis

For data analysis, we entered, organized, tabulated, and analyzed using the standard computer program Statistical Package for the Social Sciences (SPSS) version 23.0 (IBM SPSS Statistics, Armonk, NY, USA). Qualitative data were expressed as frequency and percentage. We used the chi-squared test (x^2^) and Fisher’s exact test to assess the relationship between two or more qualitative variables, and logistic regression analysis to find an association between dependent and independent variables, with the significance level set at P value < 0.05.

Ethical considerations

Formal approval was sought from the ethics committee of Qassim University of Al-Qassim, Saudi Arabia. Data were collected after obtaining ethical approval from the ethical committee. Participation in the study was voluntary, and all participants were informed of the purpose of the research and the way their data were used. Informed consent was obtained. We will also ensure the privacy of the participants and the confidentiality of their data. The aim of the study and the data collection method do not involve any ethical concerns.

## Results

Characteristics of the respondents

Our study included 444 participants. Females represented more than half of our respondents (58.6%), whereas 41.4% of the participants were males. The average age of our participants was 50 ± 16.9 years (range: 10-92 years). Regarding the nationality of our participants, we found that the majority of them were Saudi Arabian. When we assessed the marital status of our respondents, we found that 65.3% were married, 18.9% were single, 11.3% were widows, and only 4.5% were divorced. Moreover, our results found that most of the participants achieved a university education level (41%). As regards the occupation of our respondents, more than one-third of the participants (37.4%) were not working, 21.8% were government employees, and 20% were retired. Lastly, our results found that the highest percentage of our participants had <5,000 Saudi riyals (SAR) monthly income (40.3%) (Table 1).

**Table 1:** Sociodemographic characteristics of the respondents (N = 444)

Variable	Category	Frequency	Percentage
Gender	Male	184	41.4%
	Female	260	58.6%
Nationality	Saudi	428	96.4%
	Non-Saudi	16	3.6%
Marital status	Single	84	18.9%
	Married	290	65.3%
	Divorced	20	4.5%
	Widow	50	11.3%
Education status	Illiterate and primary school	115	25.9%
	Middle school	39	8.8%
	High school	90	20.3%
	University	182	41%
	Postgraduate	18	4.1%
Occupation	Government employee	97	21.8%
	Not working	166	37.4%
	Military	9	2%
	Retired	89	20%
	Others	83	18.7%
Monthly income (Saudi riyal)	<5,000	179	40.3%
	5,001-10,000	132	29.7%
	10,001-15,000	90	20.3%
	>15,000	43	9.7%

DM-related characteristics

Our findings revealed that more participants had type 2 diabetes (n = 351, 79.1%) than type 1 diabetes (n = 93, 20.9%). In addition, less than half of the participants (42.3%) admitted that they had another chronic disease. Hypertension was the most commonly reported chronic disease (70.2%), followed by thyroid disorders (14.4%) and heart disease (11.7%). The highest proportion of our respondents suffered from diabetes for more than 15 years (29.1%), and to a lesser extent, 23.9% of them had the disease for 6-10 years.

The majority of our respondents (75.2%) demonstrated that they have a family history of diabetes mellitus and 24.8% did not. Concerning the presence of diabetes complications, peripheral neuropathy was the most frequent diabetes complication, which is reported by more than half of the participants (53.2%), followed by diabetic retinopathy (34.5%), diabetic nephropathy (12.4%), coronary heart diseases (12.2%), and cerebrovascular diseases (6.8%). Furthermore, more than half of the respondents used oral hypoglycemic medication, and 41.7% used insulin on regular basis (Table 2).

**Table 2:** Diabetes mellitus-related characteristics (N = 444)

Variable	Category	Frequency	Percentage
Type of diabetes	Type 1	93	20.9%
	Type 2	351	79.1%
Do you have any other chronic diseases?	Yes	188	42.3%
	No	256	57.7%
Chronic disease (n = 188)	Hypertension	132	70.2%
	Thyroid disorders	27	14.4%
	Dyslipidemia	15	8%
	Asthma	6	3.2%
	Rheumatoid	5	2.7%
	Heart disease	22	11.7%
	Kidney disease	6	3.2%
	Epilepsy	4	2.1%
	Other	20	10.6%
Duration of diabetes mellitus	1-2 years	50	11.3%
	3-5 years	72	16.2%
	6-10 years	106	23.9%
	11-15 years	87	19.6%
	>15 years	129	29.1%
Family history of diabetes mellitus	Yes	334	75.2%
	No	110	24.8%
Presence of diabetes complications	Peripheral neuropathy	236	53.2%
	Diabetic nephropathy	55	12.4%
	Diabetic retinopathy	153	34.5%
	Coronary heart diseases	54	12.2%
	Cerebrovascular diseases	30	6.8%
What are the diabetic medication do you use regularly?	Oral hypoglycemic medication	259	58.3%
	Insulin	185	41.7%

Prevalence of CAM usage among diabetic patients and their beliefs in CAM

Our results revealed that the prevalence of CAM usage was 29.1%. When we asked about the reasons for not using CAM, about one-fourth of participants (24.1%) revealed that they were afraid of side effects, 23.5% of respondents did not believe in CAM, 17.5% of them never heard of it, and 16.2% of them reported that they did not use CAM because their doctor did not prescribe it.

Fortunately, our findings demonstrated that the majority of the participants did not believe that antidiabetic drugs could be replaced by herbs. However, more than half of the respondents believed that herbs could treat diabetes (53.5%), and 68.2% of CAM users were satisfied with herbs’ effects in controlling diabetes. Furthermore, 56.6% believed that herbs have side effects. Regarding the sources of information on herbal medicines, we found that more than half of the respondents (57.4%) obtained information from friends, relatives, and neighbors, 37.2% stated that their sources of herbal medicines were family beliefs, and 34.9% received the information from the media.

When assessing the reasons for using CAM, we found that almost half of CAM users (48.8%) believed in the advantages of CAM practices. About one-third of the respondents used CAM because of its accessibility and availability, and another one-third were looking for another solution. In addition, most of the participants (68.2%) expected CAM to reduce blood glucose levels, more than one-third of the respondents expected CAM to make them have better health status, and almost a third of respondents expected CAM to prevent the progression of diabetes (34.1%) (Table 3).

**Table 3:** Prevalence of complementary and alternative medicine usage among diabetic patients and their beliefs of complementary and alternative medicine (N = 444) CAM: complementary and alternative medicine

Prevalence of CAM usage (N = 444)		Number (%)
Do you use any CAM for diabetes currently or since diagnosis?	Yes	129 (29.1%)
	No	315 (70.9%)
Why haven’t you used CAM? (n = 315)	I’ve never heard of it.	55 (17.5%)
	I’m afraid of the side effects.	76 (24.1%)
	I don’t believe in it.	74 (23.5%)
	My doctor didn’t prescribe it.	51 (16.2%)
	I don’t want an additional burden.	19 (6%)
	No one advised me.	30 (9.5%)
	Others	10 (3.2%)
Beliefs of CAM among CAM users (n = 129)		
Do you believe that antidiabetic drugs could be replaced by herbs?	Yes	38 (29.5%)
	No	91 (70.5%)
Do you believe that herbs can treat diabetes?	Yes	69 (53.5%)
	No	60 (46.5%)
Do you believe that herbs can interact with drugs’ effects?	Yes	64 (49.6%)
	No	65 (50.4%)
Are you satisfied with herbs’ effects to control diabetes?	Yes	88 (68.2%)
	No	41 (31.8%)
Do you believe that herbs have no side effects?	Yes	56 (43.4%)
	No	73 (56.6%)
Sources from which you obtain information on herbal medicines	Media	45 (34.9%)
	Friends, relatives, and neighbors	74 (57.4%)
	Family beliefs	48 (37.2%)
	Internet	38 (29.5%)
	Health practitioner	14 (10.9%)
	Personal choice	23 (17.8%)
Why do you use CAM?	Looking for another solution	43 (33.3%)
	Believe in the advantages of CAM practices	63 (48.8%)
	CAM is accessible and available	44 (34.1%)
	Lost hope with conventional therapy	15 (11.6%)
	Others	6 (4.7%)
What was your expectation when you were using CAM?	Prevent the progression of the diabetes	44 (34.1%)
	Complete cure of the disease	19 (14.7%)
	Weight loss	23 (17.8%)
	Lower blood glucose level	88 (68.2%)
	Better health status	47 (36.4%)
	No expectations	3 (2.3%)

Use of complementary and alternative medicine

**Figure 1:**
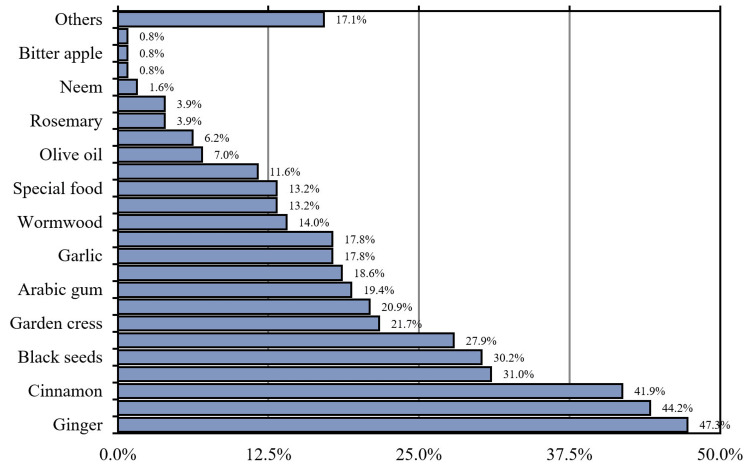
Herbal medicines our participants used (n = 129)

When we asked our respondents about the herbal medicines they used, we found that ginger was the most commonly used herbal medicine by our participants (47.3%), followed by vitamins and minerals (44.2%), cinnamon (41.9%), myrrh (31%), black seeds (30.2%), fenugreek (27.9%), garden cress (21.7%), and honey (20.9%) (Figure 1).

**Figure 2:**
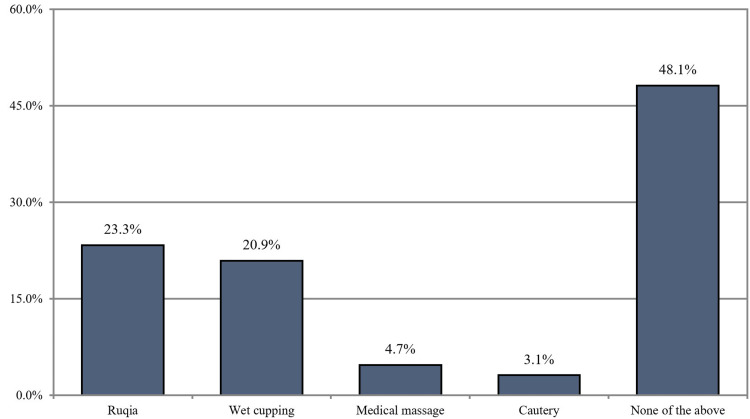
Other complementary and alternative medicine our participants used (n = 129)

Additionally, we reported that Ruqia was used by 23.3% of our respondents. Of the participants, 20.9% used wet cupping, 4.7% used medical massage, and 3.1% used cautery as complementary and alternative medicine (Figure 2).

We found that 38% of CAM users used herbal products regularly. As regards the frequency of using herbal products, 29.5% of the respondents used herbal medicine weekly and 21.7% used it daily. On the other hand, 48.8% used herbal products less frequently. Concerning the duration of use, we found that the majority of the respondents used herbal products for years (28.7%).

Moreover, our findings demonstrated that the majority of our participants (75.2%) admitted that they used CAM for other medical conditions or for their general health. Of the participants, 69.8% revealed that they did not suffer from any side effects from CAM. In addition, almost half of the respondents regard CAM as a product of limited usefulness. Most of the participants (76.7%) admitted that they told their doctor about using herbal products to control diabetes. Of the respondents, 74.4% stated that they would recommend the use of CAM treatment to other diabetic patients, and 51.6% considered using CAM in the future (Table 4).

**Table 4:** Use of CAM among CAM users (n = 129) CAM: complementary and alternative medicine

Variable		Number (%)
Do you use herbal products regularly?	Yes	49 (38%)
	No	80 (62%)
How often do you use herbal products?	Daily	28 (21.7%)
	Weekly	38 (29.5%)
	Less frequently	63 (48.8%)
Duration of use	Days	25 (19.4%)
	Weeks	33 (25.6%)
	Months	34 (26.4%)
	Years	37 (28.7%)
Do you use CAM for any other medical conditions or for your general health?	Yes	97 (75.2%)
	No	32 (24.8%)
Have you suffered from any side effects from CAM?	Yes	10 (7.8%)
	No	90 (69.8%)
	Not sure	29 (22.5%)
How do you assess the usefulness of CAM?	Very useful	37 (28.7%)
	Of limited usefulness	65 (50.4%)
	Not sure/unable to assess	23 (17.8%)
	Not useful at all	4 (3.1%)
Do you tell your doctor about using herbal products to control diabetes?	Yes	99 (76.7%)
	No	30 (23.3%)
Would you recommend the use of CAM treatment to other diabetic patients?	Yes	96 (74.4%)
	No	33 (25.6%)
Would you consider using CAM in the future?	Yes	229 (51.6%)
	No	215 (48.4%)

**Figure 3:**
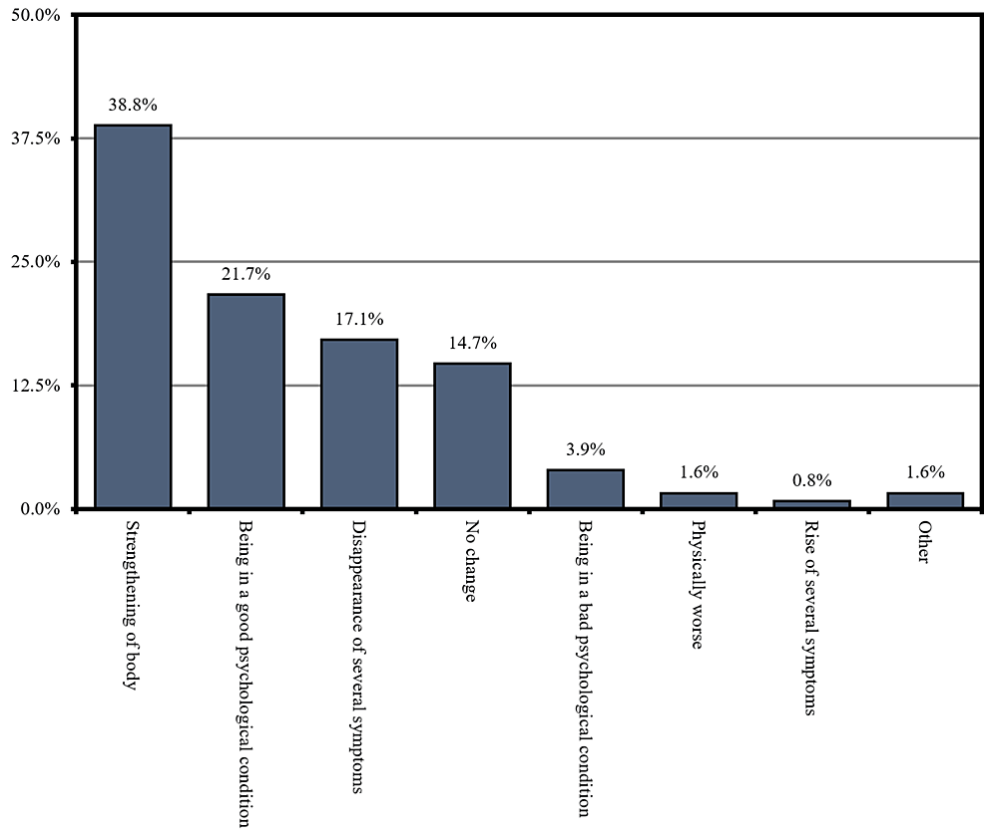
What was your feeling after using CAM (n = 129)? CAM: complementary and alternative medicine

When we asked our participants about their feeling after using CAM, 38.8% of them clarified that CAM is strengthening the body, and 21.7% stated that they have been in good psychological health. Moreover, 17.1% revealed that using CAM led to the disappearance of several symptoms (Figure 3).

Factors associated with the use of complementary and alternative medicine

Our results found that there was a significant association between using CAM for diabetes and gender; females use CAM significantly higher than males (P value = 0.008). Moreover, there was a significant association between marital status and income in relation to using CAM for diabetes (P value = 0.011 for both). The use of CAM was significantly higher in married participants and participants with a monthly income of 10,001-15,000 SAR. Lastly, we did not find any significant association between using CAM for diabetes and nationality, education status, occupation, and having a chronic disease (Table 5).

**Table 5:** Factors associated with the use of complementary and alternative medicine CAM: complementary and alternative medicine

Variable	Category	Use of CAM for diabetes		P value
		Yes	No	
Gender	Male	41 (22.3%)	143 (77.7%)	0.008
	Female	88 (33.8%)	172 (66.2%)	
Nationality	Saudi	122 (28.5%)	306 (71.5%)	0.259
	Non-Saudi	7 (43.8%)	9 (56.3%)	
Marital status	Single	12 (14.3%)	72 (85.7%)	0.011
	Married	96 (33.1%)	194 (66.9%)	
	Divorced	6 (30%)	14 (70%)	
	Widow	15 (30%)	35 (70%)	
Education status	Illiterate and primary school	38 (33%)	77 (67%)	0.300
	Middle school	14 (35.9%)	25 (64.1%)	
	High school	20 (22.2%)	70 (77.8%)	
	University	50 (27.5%)	132 (72.5%)	
	Postgraduate	7 (38.9%)	111 (61.1%)	
Occupation	Government employee	29 (29.9%)	68 (70.1%)	0.241
	Not working	57 (34.3%)	109 (65.7%)	
	Military	1 (11.1%)	8 (88.9%)	
	Retired	22 (24.7%)	67 (75.3%)	
	Others	20 (24.1%)	63 (75.9%)	
Monthly income (Saudi riyal)	<5,000	39 (21.8%)	140 (78.2%)	0.011
	5,001-10,000	39 (29.5%)	93 (70.5%)	
	10,001-15,000	37 (41.1%)	53 (58.9%)	
	>15,000	14 (32.6%)	29 (67.4%)	
Having chronic disease	Yes	58 (30.9%)	130 (69.1%)	0.475
	No	71 (27.7%)	185 (72.3%)	

Predictors for the prevalence of CAM usage for diabetes

The results revealed that the odds of CAM usage increased among married participants by 4.12-folds and among divorced participants by 9.75-folds when compared with single participants, and it is statistically significant (P value = 0.039 and 0.008, respectively). However, the effect of marital status on the odds of CAM usage did not reach the significance level (P = 0.068). Other variables did not have a significant effect on the odds of CAM usage among diabetic patients. Further information is provided in Table 6.

**Table 6:** Predictors for the prevalence of CAM usage for diabetes CAM: complementary and alternative medicine, CI: confidence interval

Variable	B	Odds ratio	95% CI for odds ratio		P value
			Lower	Upper	
Age	-.005				0.725
Gender					0.312
Male		1			
Female		1.469	0.697	3.099	
Nationality					0.773
Saudi		1			
Non-Saudi		0.787	0.155	4.004	
Marital status					0.068
Single		1			
Married		4.117	1.077	15.743	0.039
Divorced		9.748	1.831	51.907	0.008
Widow		5.130	0.932	28.221	0.060
Education status					0.415
Primary school		1			
Middle school		2.277	0.822	6.311	0.114
High school		1.474	0.504	4.315	0.479
University		1.966	0.691	5.590	0.205
Postgraduate		3.768	0.693	20.481	0.125
Occupation					0.378
Government employee		1			
Not working		2.184	0.813	5.864	0.121
Military		0.000	0.000	.	0.999
Retired		0.816	0.287	2.322	0.703
Others		1.653	.567	4.824	0.357
Monthly income (Saudi riyal)					0.403
<5,000					
5,001-10,000		1.308	0.582	2.940	0.515
10,001-15,000		1.849	0.755	4.529	0.179
>15,000		0.750	0.197	2.849	0.673
Having chronic disease					0.653
No		1			
Yes		1.157	0.613	2.184	

## Discussion

The present study aimed to know the habits, traditions, and beliefs associated with the use of complementary and alternative medicine among diabetic patients in Al-Qassim, Saudi Arabia. It is estimated that 80% of the world’s population uses CAM for primary healthcare, making it more common to use it as an alternative to conventional therapy for many diseases [16]. One of the nations with strong cultural and traditional values is Saudi Arabia, and for many years, people in Saudi Arabia have used CAM as a folk remedy to treat a variety of illnesses [17].

The current study revealed that the prevalence of CAM usage was 29.1%. Several studies showed variations in results regarding the use of CAM among type 2 diabetes patients. Our results were lower than the studies conducted in other parts of Saudi Arabia, such as in Riyadh at 84.6%, Jazan at 34%, and Jeddah at 64% [18-20]. Another study that was conducted in UAE reported that 39.3% of participants were CAM users since diagnosis [12]. When compared to our study findings, studies in the UK and Germany revealed a lower prevalence of CAM use, at 17% and 18.4%, respectively [21,22]. These variations in cultural perceptions of CAM use, along with differences in the study design and definition of CAM used by various studies, could account for these differences in the prevalence of CAM use by region.

Our results found that more than half of the participants (56.6%) believed that herbs have side effects, and 68.2% of them were satisfied with herbs’ effects to control diabetes. This is in accordance with an older study that stated that 64.5% of respondents noticed an improvement in blood sugar levels while using herbs. The same study in Jeddah showed that 54.2% of respondents experienced no side effects using herbs [20]. Moreover, our findings clarified that friends, relatives, and neighbors were primary sources of information regarding CAM. This is in agreement with another study that demonstrated that mass media (e.g., TV, newspapers, and radio) and family, relatives, and friends represented the main sources of CAM knowledge (46.5% and 46.3%, respectively) [18]. This suggests that the respondents’ understanding of trustworthy and legitimate sources for information about CAM is lacking.

When analyzing the motivations for using CAM, we discovered that nearly half of the participants (48.8%) thought CAM practices had benefits. Due to CAM’s availability and accessibility, about one-third of respondents used it. This is supported by a prior study, which found that most respondents cited affordability, accessibility, acceptability, and effectiveness as the main reasons they chose to use complementary and alternative medicine (CAM), as it is widely accessible and available throughout most of Saudi Arabia [19]. Another study that was carried out in Sudan revealed that accessibility and affordability were the suggested reasons for using herbal remedies [23].

Ginger, vitamins and minerals, cinnamon, and Ruqia were the most frequently used CAM among our participants. This may be related to habits and beliefs among the Saudi population and religious background. Compared to the present study, two studies that were done in Jordan and Palestine found that the most commonly used herb is fenugreek [24,25]. In addition, most of the participants (76.7%) admitted that they counsel their doctor about using herbal products to control diabetes. These findings were higher than the results of two studies done in Saudi Arabia [18,19].

Additionally, our results found that there was a significant association between using CAM for diabetes and gender. Females use CAM more significantly than males. This is in line with other studies in Riyadh and Thailand [18,26]. This may be explained by females finding it difficult to visit doctors, by women spending a lot of time at home because of their love of media, by women’s attachment to their families, friends, and loved ones, and by the prevalence of herbs in households. However, a study conducted in the UAE revealed that males made up the majority of CAM users [12]. Our findings support an earlier study from Lebanon that found that CAM use was significantly correlated with marital status [27].

Limitations

A self-interviewing questionnaire was used in this study. As a result, the outcome is susceptible to recall bias. Furthermore, courtesy bias may be present in the interviewed patients, who may be prone to changing their answers for any reason.

The study was limited also to the residents of the Al-Qassim region in Saudi Arabia. As a result, the results may not accurately reflect the Saudi population as a whole.

## Conclusions

According to our research, CAM use among diabetes patients in the Al-Qassim region was found to be relatively common and high. The use of CAM was significantly correlated with gender, marital status, and monthly income. The prevalence of type 2 diabetes (79.1%) was higher than that of type 1 diabetes (20.9%). Also, the most commonly used herb was ginger, followed by vitamins and minerals and cinnamon. Patients with diabetes need to be made aware of the value of telling their doctors about their use of CAMs. Diabetes patients’ knowledge of herbal use may be improved through awareness programs administered by physicians and nurses using various educational strategies for counseling and educating patients in order to achieve good therapeutic outcomes. To protect patients’ health and well-being, health policymakers and decision-makers should pay special attention to facilitating proper regulation and integration of complementary and alternative medicine (CAM) into conventional medicine.
